# Potential Role of Estrogen Receptor Beta as a Tumor Suppressor of Epithelial Ovarian Cancer

**DOI:** 10.1371/journal.pone.0044787

**Published:** 2012-09-06

**Authors:** Carine Bossard, Muriel Busson, David Vindrieux, Françoise Gaudin, Véronique Machelon, Madly Brigitte, Carine Jacquard, Arnaud Pillon, Patrick Balaguer, Karl Balabanian, Gwendal Lazennec

**Affiliations:** 1 Institut National de la Santé et de la Recherche Médicale (INSERM), U844, University of Montpellier I, Montpellier, F-34091, France; 2 Laboratory of Excellence in Research on Medication and Innovative Therapeutics (LERMIT), Institut National de la Santé et de la Recherche Médicale (INSERM), UMR_S996, Univ. Paris-Sud, Clamart, France; 3 Institut de Recherche en Cancérologie de Montpellier (IRCM), Montpellier, France; University of Nebraska Medical Center, United States of America

## Abstract

Ovarian cancer is the gynecological cancer exhibiting the highest morbidity and improvement of treatments is still required. Previous studies have shown that Estrogen-receptor beta (ERβ) levels decreased along with ovarian carcinogenesis. Here, we present evidence that reintroduction of ERβ in BG-1 epithelial ovarian cancer cells, which express ERα, leads *in vitro* to a decrease of basal and estradiol-promoted cell proliferation. ERβ reduced the frequency of cells in S phase and increased the one of cells in G2/M phase. At the molecular level, we found that ERβ downregulated total retinoblastoma (Rb), phosphorylated Rb and phospho-AKT cellular content as well as cyclins D1 and A2. In addition, ERβ had a direct effect on ERα, by strongly inhibiting its expression and activity, which could explain part of the anti-proliferative action of ERβ. By developing a novel preclinical model of ovarian cancer based on a luminescent orthotopic xenograft in athymic Nude mice, we further revealed that ERβ expression reduces tumor growth and the presence of tumor cells in sites of metastasis, hence resulting in improved survival of mice. Altogether, these findings unveil a potential tumor-suppressor role of ERβ in ovarian carcinogenesis, which could be of potential clinical relevance for the selection of the most appropriate treatment for patients.

## Introduction

The single epithelial cell layer that surrounds ovaries is currently believed to be one of the sources of preneoplastic lesions leading rise to epithelial ovarian tumors, which represent the vast majority of ovarian cancers [1]. Epithelial ovarian cancer (EOC) is the seventh most common cancer. However, it remains the fourth most deadly one because it is difficult to diagnose at early stages and, hence, to treat [2]. Either classified on morphological categories (i.e., serous, mucinous, endometrioid, and clear cells) based on histological criteria and resemblance to epithelial components of the normal reproductive tract, or more recently, classified as low- or high-grade tumors [2], EOC is a complex disease for which the etiology is poorly understood. Novel markers and targets for therapies are thus urgently needed.

Ovary is the main organ of production of estrogens, which mainly impact on the growth, differentiation and function of reproductive tissues [3]. Through their mitogenic action, estrogens play roles in ovarian carcinogenesis. Several studies have highlighted an increased risk of ovarian cancer in patients receiving long-term estrogen replacement therapy [4], [5], [6], [7], while patients treated with oral contraception combining estrogens and progestins showed a reduced risk of developing an ovarian cancer [8], [9]. Estrogen action is mediated by two receptors, ERα and ERβ, two transcription factors of a large family of nuclear receptors [10], [11]. About 40 to 60% of ovarian cancers express ERα [12], but it is intriguing to notice that only a small proportion of them will benefit from anti-estrogen therapy [13]. The role of ERβ in the ovarian biology remains poorly understood, but it seems to be different from that of ERα [14]. *ERβ* knock-out animals (βERKO) are subfertile, producing fewer litters and pups upon superovulation induction [15], [16]. The ovaries of βERKO animals contain fewer large antral follicles and corpus luteum compared to wild-type littermates, which is concomitant with lower levels of estradiol produced [17] and a reduced expression of key genes involved in ovary function such as aromatase (*Cyp19a1*), LH receptor (*Lhcgr*), and prostaglandin synthase 2 (*Ptgs2*) [18].

Several studies have unraveled a potential role for ERβ in EOC. In particular, ERβ levels were lower in ovarian tumors compared to normal tissues [19], [20], [21], [22]. Moreover, the loss of ERβ expression could correlate with a shorter overall survival of ovarian cancer patients [23]. ERβ levels are also associated with metastatic lymph node status [24]. A polymorphism (rs127572) of the *ERβ* gene has also been identified recently and shown to be associated with an increased risk of developing an ovarian cancer [25]. However, it is still unknown whether this polymorphism affects the expression of ERβ. The intracellular location of ERβ in tumor cells seems to be important. Indeed, a recent study has shown that ERβ was localized in the cytoplasm of tumor cells, while it was mainly nuclear in normal epithelial cells [26]. In addition, cytoplasmic expression of ERβ was correlated to a poor outcome for patients with advanced serous ovarian cancer [14]. These findings, combined with the aforementioned clinical correlations between ERβ and patient survival, lead us to hypothesize that ERβ is a critical factor in ovarian tumor progression and to delineate the precise contribution of this receptor in the molecular pathways underlying EOC carcinogenesis.

For this purpose, we used BG-1 cells as a cellular model and took advantage of an orthotopic xenograft mouse model we have developed. BG-1 cell line is a human EOC cell line derived from a solid primary tumor tissue from a patient with stage III, poorly differentiated ovarian adenocarcinoma [27]. These cells express ERα and are sensitive to estrogens in terms of proliferation [21], [28]. Experimental models of ovarian carcinogenesis are essential to understand the molecular mechanisms involved in the development of the disease but also to evaluate the efficacy of novel therapeutic drugs [29]. Several models have been developed, including different xenograft and transgenic models, none being fully satisfactory. The xenograft models that are currently used are either intraperitoneal, subcutaneously or orthotopically intrabursal in the ovary. Only few reports describe orthotopic xenograft. Nevertheless, orthotopic cell implantation can be perceived as more physiological, as the cancer cells are directly inoculated in the ovarian environment and can lead to metastasis. Therefore, to investigate the role of ERβ in EOC carcinogenesis, we chose to take advantage of an orthotopic xenograft mouse model based on the use of luciferase (Luc)-expressing human epithelial ovarian cancer BG-1 cells.

We show here that reintroduction of ERβ in BG-1 cells using an adenovirus leads *in vitro* to an inhibition of both basal and estradiol-induced cell proliferation. ERβ exerts its anti-proliferative action through a reduction of the frequency of cells in S phase, an increase of cells in G2 phase, along with an altered expression of cell cycle regulators. At the molecular level, ERβ was able to repress the expression, the activity and the signaling of ERα, and thus to block its proliferative action. Moreover, ERβ was able to strongly reduce the development of orthotopic ovarian xenograft as well as the presence of tumor cells the sites of metastasis, leading to an increased survival of the mice. Altogether, these findings support a role for ERβ as tumor-suppressor in EOC carcinogenesis.

## Results

**Figure 1:**
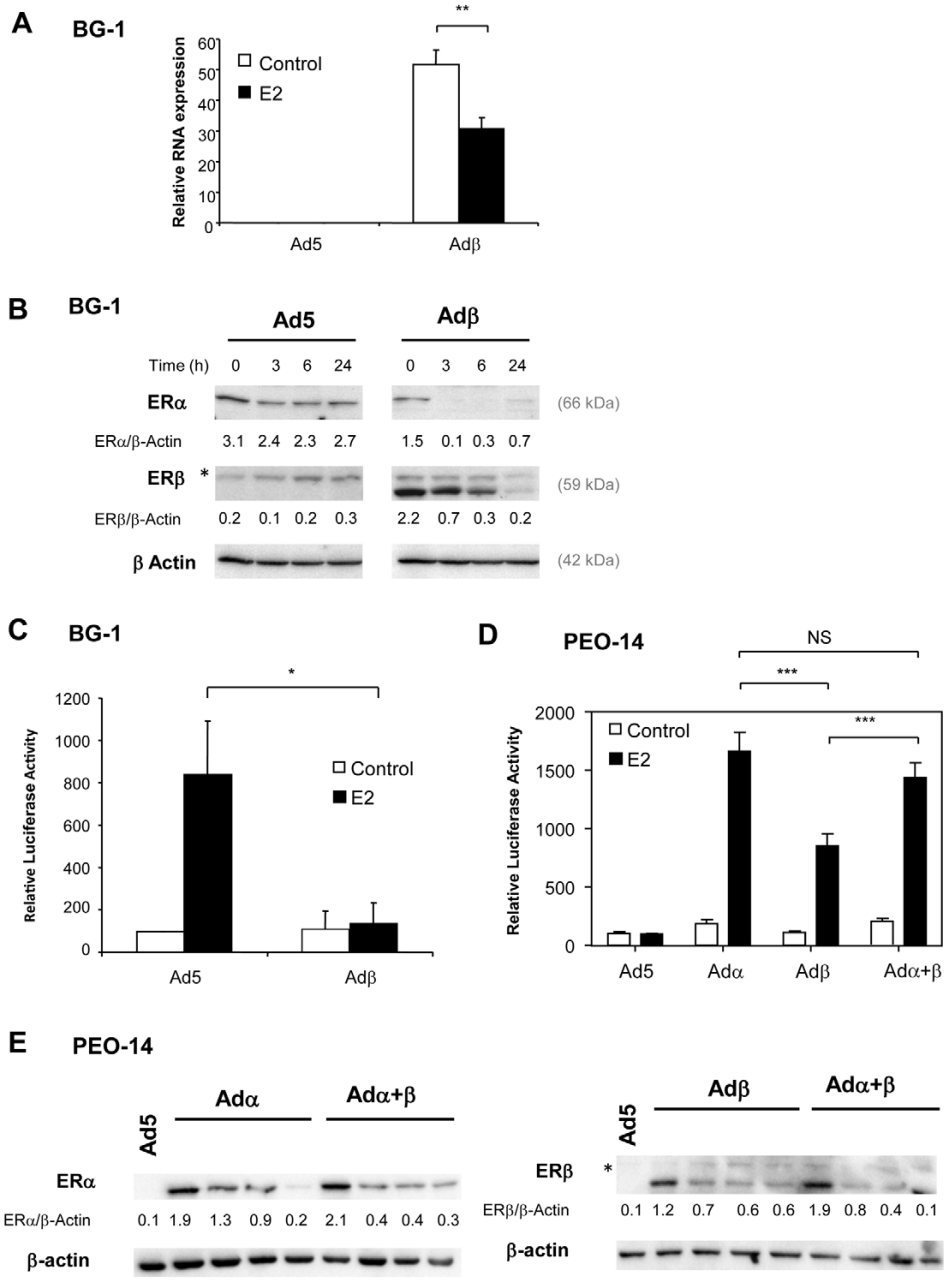
Expression levels and transcriptional activity of ERβ in BG-1. A. BG-1 cells were infected with Ad5 or Adβ adenoviruses and treated for 24h with control vehicle ethanol (Control) or E2 10^−8^M (E2). The expression of ERβ and rS9 reference gene was measured by real-time PCR. Results represent the mean ± SD of ERβ expression normalized by rS9 of 3 independent experiments. Measurements of Adβ and Adβ+E2 groups were compared by unpaired Student's *t* test. ** p<0.001. **B.** Proteins were extracted from cells infected in the same conditions as in A and treated for 0, 3, 6 or 24h with E2 10^−8^M (E2). Levels of ERα and ERβ were analyzed by western blot. Actin was used as a loading control. The upper band of ERβ blot labeled with a star corresponds to aspecific staining. **C.** Transcriptional activity of ERβ. BG-1 cells were infected with Ad5 or Adβ adenoviruses and transfected with ERE2-TK-Luc reporter along with β-galactosidase reporter. The cells were treated with ethanol as vehicle (Control) or E2 (10−8M) for 24h. Results show relative Luc activities (% of values of Ad5-infected cells without E2) ± SD after normalization with β-gal activity (3 independent experiments). Measurements of Ad5+E2 and Adβ+E2 groups were compared by unpaired Student's *t* test. * p<0.05. **D.** PEO14 cells were infected with Ad5, Adα, Adβ or the combination of Adα and Adβ adenovirus and transfected with ERE2-TK-LUC reporter along with β-galactosidase reporter. Cells were treated with control vehicle (Control) or E2 (10−8M) for 24h. Results show relative luciferase activities (% of values of Ad5 infected cells without E2) ± SD after normalization with β-gal activity (3 independent experiments). Measurements of Adα, Adβ and Adα+β groups were compared by unpaired Student's *t* test. *** p<0.001. NS: non significant. **E.** Proteins from PEO14 cells infected in the same conditions as in D and treated with vehicle ethanol (Control) or E2 10^−8^M (E2) for 3, 6 or 24h were analyzed by western blot with antibodies against ERα and ERβ. Actin was used as a loading control. The upper band labeled with a star in right panel corresponds to aspecific staining.

Previous reports have shown that ERβ is weakly expressed in EOC tissues and derived cell lines compared to normal tissue [11]. We took advantage of the human EOC cell line BG-1, which expresses endogenous levels of ERα and is sensitive to estrogens [27]. Here, we first confirm that BG-1 cells display low steady-state levels of ERβ products, i.e. mRNA and proteins (Fig. 1A, B). The next step towards assessing the role of ERβ in ovarian carcinogenesis was to restore its expression in ovarian cancer cells. Thus, BG-1 cells were infected with a backbone (Ad5) or human *ERβ* (Adβ) encoding adenovirus [30], [31]. *ERβ* overexpression was indeed obtained in Adβ-infected cells as validated by real-time PCR and Western blot analyses (Fig. 1A, B). Interestingly, ERβ levels were strongly down-regulated by estradiol (E2) both at RNA and protein levels. Moreover, in the absence of ERβ, ERα levels were also down-regulated by E2, although at a lesser extent. When ERβ was introduced in the cells, the basal expression level of ERα in the absence of E2 was reduced and by half. The presence of ERβ strongly diminished ERα levels in the presence of E2 (more than 15 fold decrease in 3 h), suggesting that ERβ enhances the degradation of ERα. This is likely due to proteasome-dependent degradation of ERα and ERβ proteins [32]. The effects of ERβ overexpression on estrogen responsiveness were then investigated. We analyzed the ability of ERβ to transactivate a synthetic luciferase reporter sensitive to estrogens in BG-1 cells. Endogenous ERα was able to activate the reporter in the presence of E2 (Fig. 1C). ERβ strongly repressed the activity of ERα in response to E2, suggesting that in the presence of ERα, ERβ behaves rather as a repressor than an activator of estrogen signaling. To ensure of the functionality of the receptor produced, we also checked the activity of ERβ in the EOC cell line PEO14 [33] that is reported to express low levels of ERα (Fig. 1E) and hence does not respond to estrogens [34]. Both ERα and ERβ were able to stimulate an estrogen-sensitive reporter upon E2 exposure, even though ERβ was a little bit less active than ERα (Fig. 1D). Therefore, in the absence of ERα, ERβ retains the ability to transactivate estrogen signaling pathways. When ERα and ERβ were coexpressed in PEO-14 cells, the activity of the reporter in the presence of E2 was similar to the one of ERα alone. This suggests that in the ER-negative PEO-14 cell line, ERβ cannot affect ERα activity. Western blot experiments in PEO-14 cells show that when ERα and ERβ were expressed separately, their levels were strongly decreased in the presence of E2 (Fig. 1E). When ERα and ERβ were coexpressed, the degradation of ERα in the presence of E2 was slightly increased but not in the proportion seen in BG-1 cells. For ERβ, the coexpression of ERα enhanced slightly the degradation of ERβ. Overall, these data suggest that ERα levels are not regulated in the same manner in BG-1 and PEO-14 cells, which could explain why ERα activity is not drastically reduced by the presence of ERβ in PEO-14 in comparison to BG-1 cells.

**Figure 2:**
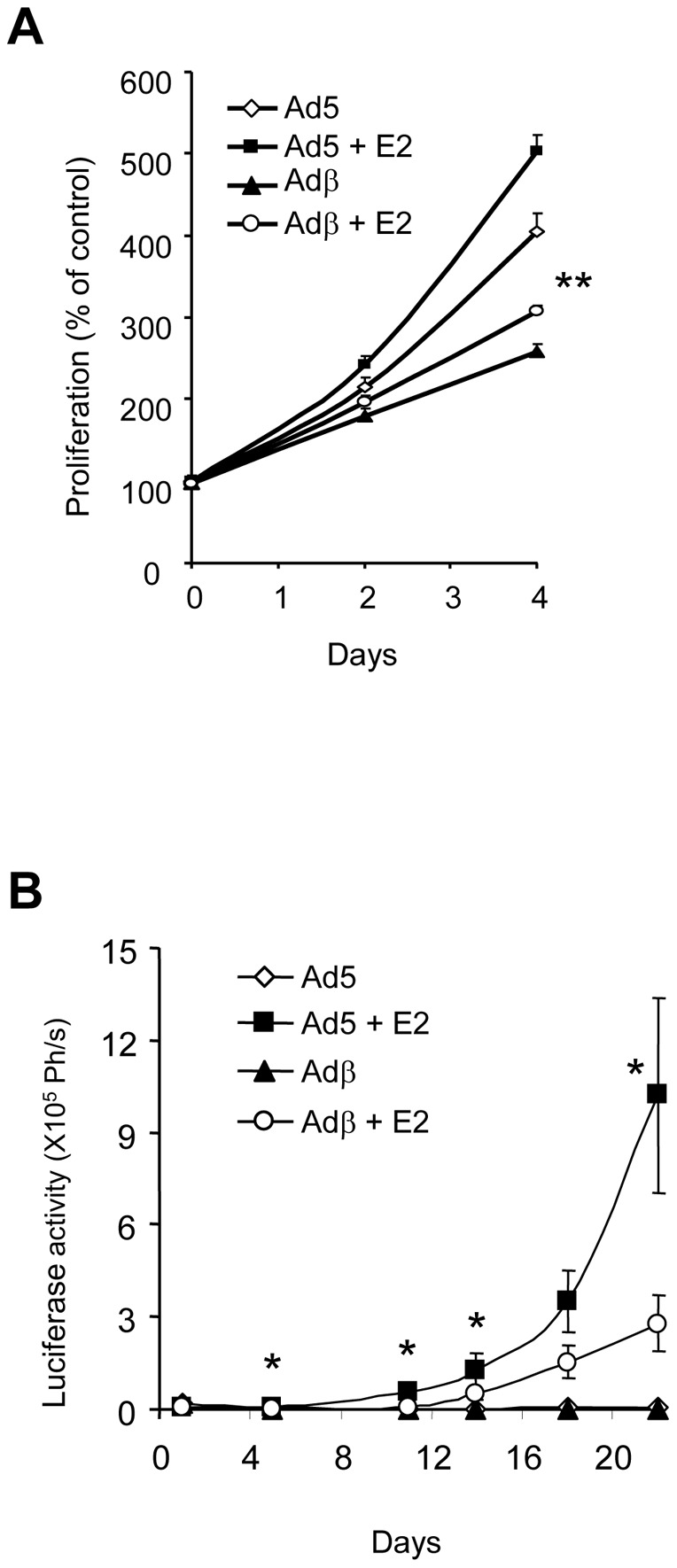
ERβ is a negative regulator of BG- cell growth. A. The growth of BG-1 cells, expressing or not ERβ, was monitored *in vitro* using a cell counter. BG-1 cells were plated in 24-well plates and cultured in the presence of vehicle or E2 (10^−8^M). Proliferation is expressed as % of control cells grown at day 0. Data represent the mean ± SD from triplicates. Measurements of Ad5+E2 and Adβ+E2 groups were compared by unpaired Student's *t* test. ** p<0.001. **B.** ERβ inhibits tumor growth in a bioluminescent subcutaneous mouse model. BG-1 cells stably expressing Luc and infected with Ad5 or Adβ adenoviruses were injected subcutaneously in ovariectomized female Nude mice. Luciferase activity was monitored for 25 days. Results are expressed in photons/s (Ph/s) and represent the mean ± SD from 8 animals. Measurements of Ad5+E2 and Adβ+E2 groups were compared by unpaired Student's *t* test. * p<0.05.

We next studied the effects of *ERβ* expression on cancer cell proliferation. BG-1 cells infected with Ad5 or Adβ adenoviruses were grown *in vitro* in the absence or the presence of E2. As expected, E2 stimulated the proliferation of control BG-1 cells (Fig. 2A). Interestingly, ERβ repressed by 50% both the basal and E2-dependent proliferation of BG-1 cells. We also observed anti-proliferative action of ERβ in ERα- and ERβ-negative PEO14 cells. However, this inhibition was not affected by E2 (Figure S2). While the *in vitro* effects of ERβ have been observed so far, very little is known of its *in vivo* action in ovarian cancer models. As a first approach to assess this point, we used a model of subcutaneous injection. To enable a sensitive, dynamic and early follow-up of tumor growth, we stably transfected BG-1 cells with a constitutive Luc reporter. Ovariectomized athymic Nude mice were implanted a pellet of cholesterol or E2 and injected with BG-1 cells infected with Ad5 or Adβ adenoviruses. BG-1 cells formed tumors only in the presence of E2 (Fig. 2B). *ERβ* expression significantly reduced by 70% the E2-promoted growth of BG-1 cells, thus supporting a potential anti-proliferative role for ERβ in EOC.

**Figure 3:**
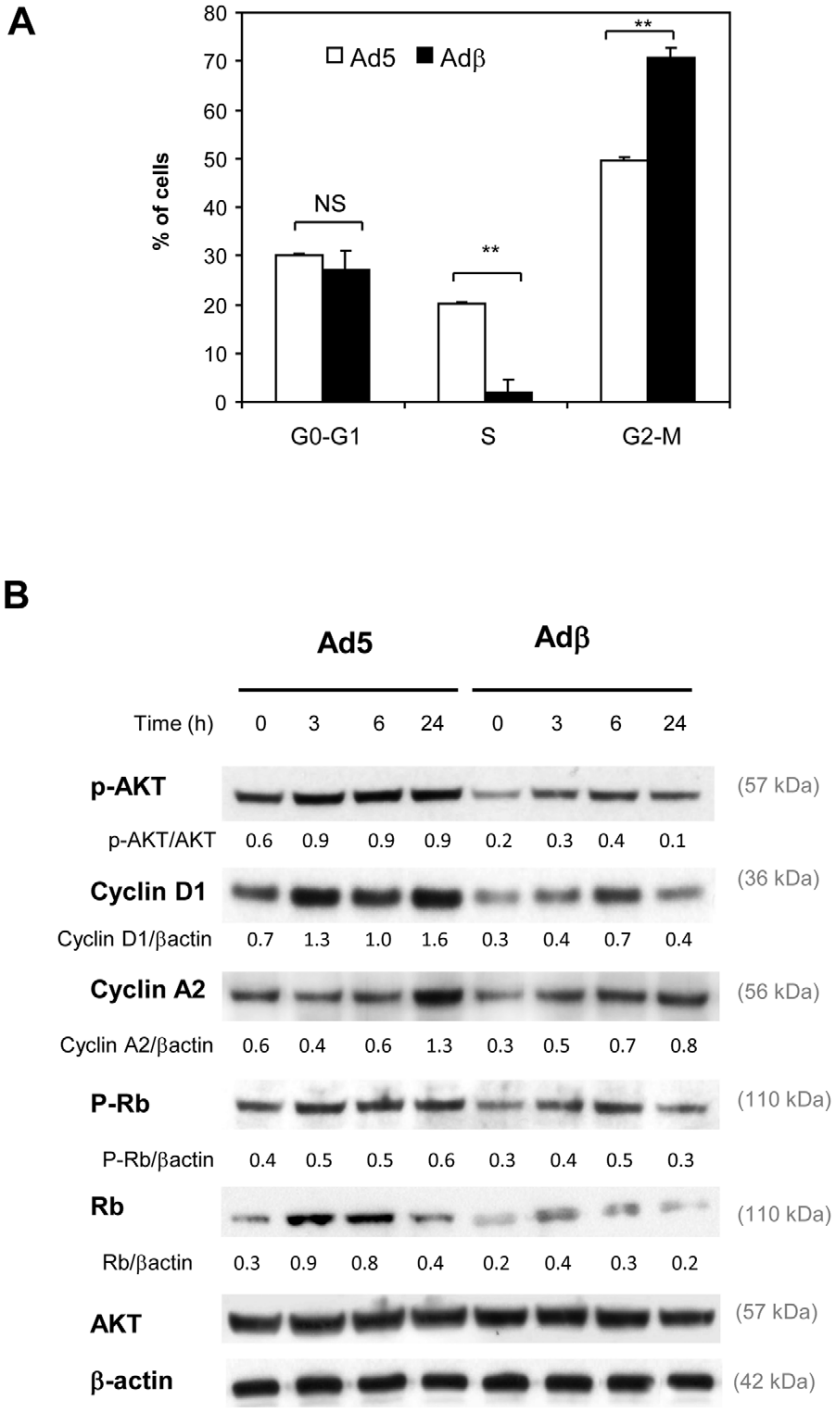
ERβ disturbs cell cycle of BG-1 cells and regulators. A. BG-1 cells were collected 24h after infection with Ad5 or Adβ adenoviruses and analyzed for cell cycle distribution. Results represent the mean ± SEM of 3 experiments. Ad5 and Adβ groups were compared by unpaired Student's *t* test. ** p<0.001. **B.** BG-1 cells were infected with Ad5 or Adβ viruses. After infection, cells were treated for 3, 6 or 24h with vehicle (ethanol, C) or 10^−8^M E2. 30 µg of protein extracts were used for Western blot. β-actin was used as a loading control. Unless specified, the ratio of target proteins over β-actin is indicated below the gels. Representative of 2 experiments.

We then explored the mechanisms responsible for the anti-tumoral action of ERβ. Cell cycle distribution was compared by flow cytometry in control and ERβ-expressing BG-1 cells (Fig. 3A). We did not detect any SubG0 peak, suggesting that no apoptosis occurred. *ERβ* expression did not modify the proportion of BG-1 cells in G0-G1 phase, but it strongly reduced that of cells in S phase. We also found that *ER*β expression triggered an increased number of cells in G2-M phase of cell cycle. The expression of cell cycle markers was next monitored by Western blot analyses (Fig. 3B). Several cell cycle regulators such as Cyclins A and D1, AKT and Rb are reported to be regulated by estrogens [35]. We observed that phosphorylation of AKT was increased upon E2 treatment in Ad5-infected BG-1 cells (Fig. 3B). *ERβ* expression led to a decrease in phospho-AKT content in both vehicle- and E2-teated cells and this occurred at 3, 6 and 24h. ERβ caused similar changes in Cyclin D1, total Rb and phosphorylated Rb expression. Indeed, the levels of cyclin D1, total Rb and phosphorylated Rb were up-regulated upon E2 treatment in Ad5 infected cells and this induction was reduced by ERβ. Cyclin A2 displayed a late induction 24h after E2 treatment and this was reduced by the presence of ERβ.

**Figure 4:**
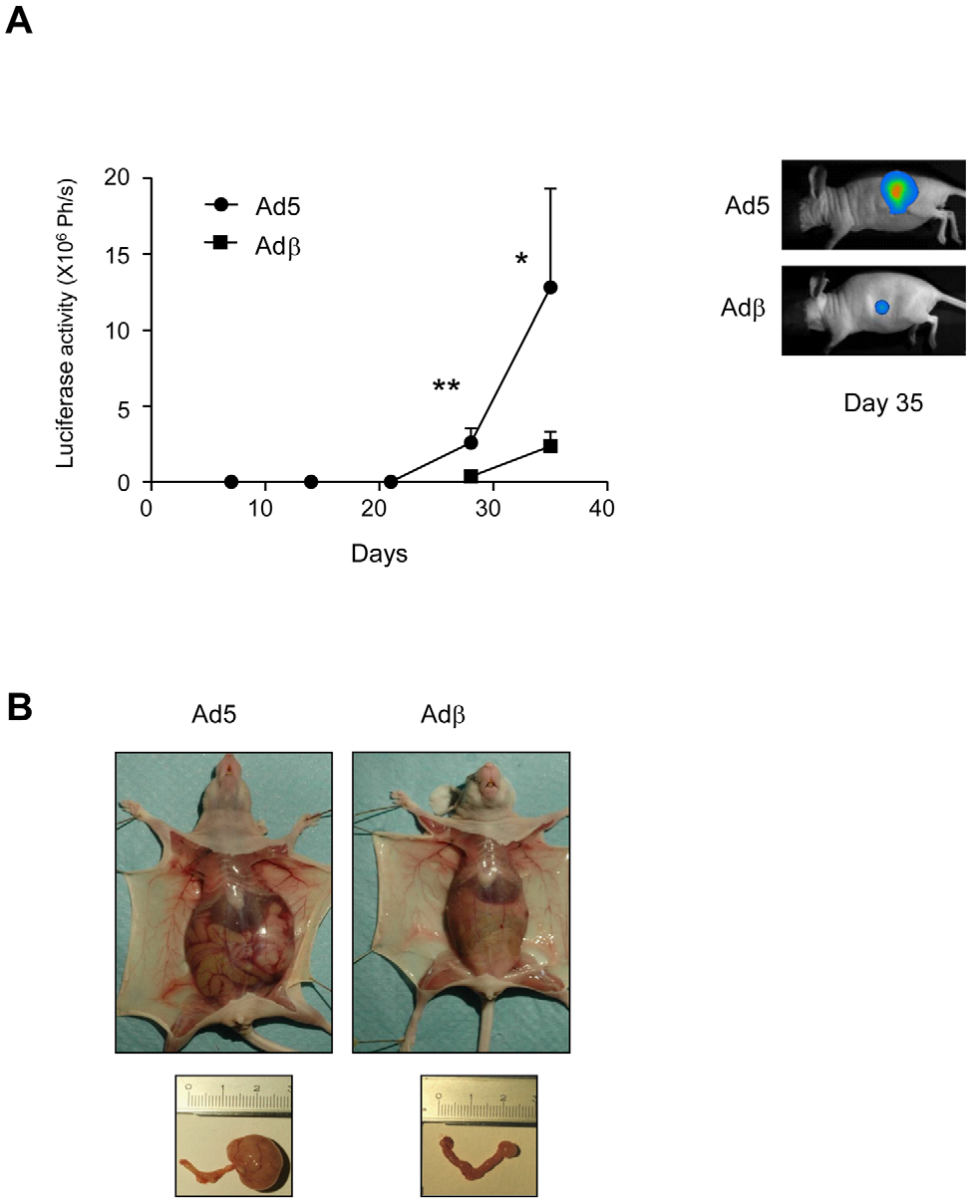
ERβ inhibits tumor growth in an orthotopic xenograft mouse model. A. BG-1-Luc cells infected with Ad5 or Adβ adenoviruses were injected in the left ovary of Nude mice. Luc activity was monitored for 35 days as described in Fig. 1C. Results are expressed in photons/s (Ph/s) and represent one representative experiment corresponding to the average ± SD of at least 5 animals per group. Mann-Whitney test was used for comparison. * p<0.05 and ** p<0.01. A representative image of day 35 is shown. **B.** Representative pictures of whole animals and genital tract of animals euthanized at day 35 are displayed.

**Figure 5:**
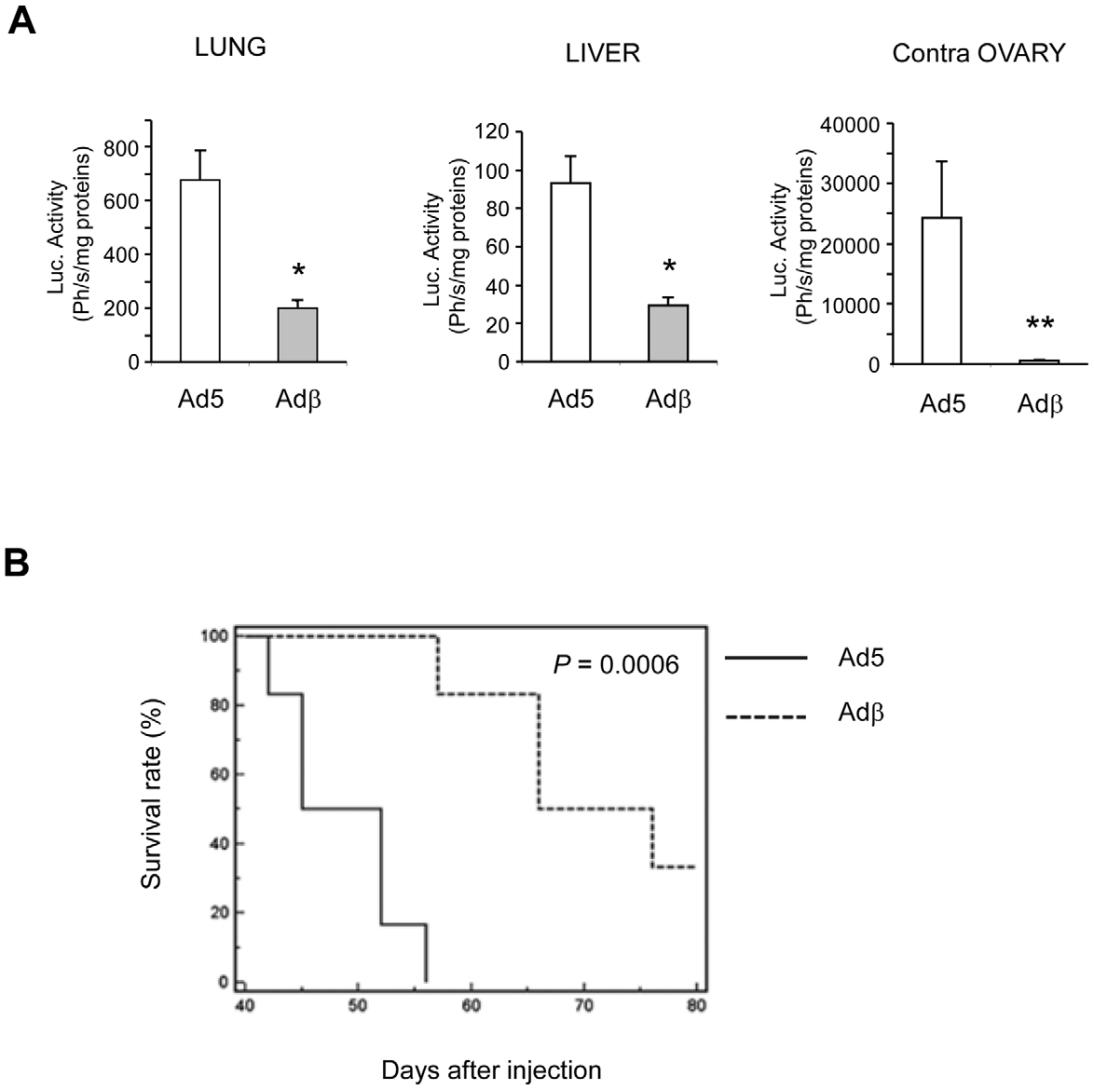
ERβ reduces metastasis and improves survival. A. Nude mice injected with BG-1-Luc cells in the same conditions as in Fig. 4. were euthanized 28 days after injection. Lung, liver and right contralateral ovary were taken and Luc activity was assayed as mentioned above. Results are expressed as Photons/s/mg of proteins and represent the mean of 5 animals ± SEM. Mann-Whitney test was used for comparison. * p<0.05 and ** p<0.01. **B.** Kaplan-meier survival curve. 10 mice were orthotopically xenografted with BG-1 cells stably expressing Luc, infected with Ad5 or Adβ adenoviruses, followed-up daily for the development of respiratory distress, limb paralysis and weight loss, and euthanized immediately if noted. P value is the one obtained in log-rank tests.

To further explore the *in vivo* role of ERβ in ovarian carcinogenesis, we set up a more physiologic model of orthotopic implantation of tumor cells in the ovary. Ad5- or Adβ-infected BG-1-Luc cells were injected in the left ovary and tumor growth was monitored by bioluminescence. As shown in Fig. 4A, *ERβ* expression significantly prevented tumor growth. When euthanized at day 28 post-injection, control mice displayed a clear increase of peritoneal volume and of tumor volume in the left ovary (Fig. 4B). In sharp contrast, the volume of both peritoneum and ovary appeared much reduced in mice injected with ERβ-expressing BG-1 cells. We next determined whether the metastatic process was affected by *ERβ* expression. To achieve this, the lung, liver and contralateral right ovary were collected. The extent of tumor cells present in these organs was estimated by measuring the Luc activity in our orthotopic model. Luciferase activity was detected in the lung, liver and contralateral ovary from mice injected with Ad5-infected BG-1 cells, (Fig. 5A). Interestingly, *ERβ* expression strongly reduced the presence of tumor cells and potentially metastasis to all these organs, suggesting that ERβ exerts a dual role on the tumor growth and dissemination. This was confirmed by *in vitro* wound healing experiments showing that *ERβ* expression decreases the motility of BG-1 cells (data not shown). Since ERβ impacted on both tumor growth and cell dissemination, we wondered whether this could enable to improve mice survival. Mice were followed-up for 80 days and daily checked for any sign of morbidity. *ERβ* expression significantly delayed death as two months after injection with Adβ-infected BG-1 cells, 50% of mice still survived (Fig. 5B). This is in strong contrast with the 100% death of control mice, which occurs in less than two months.

## Discussion

We report here that the introduction of ERβ in ovarian cancer cells displaying endogenous levels of ERα leads to a strong inhibition of *in vivo* growth and cell dissemination, mediated through the control of ERα expression and signaling.

*In vitro* proliferation experiments show a clear inhibition of the E2-dependent proliferation by ERβ in the ERα-positive ovarian cancer cell line BG-1. This is the first demonstration of an anti-proliferative action of ERβ in an ERα-positive ovarian cancer cell line. Moreover, ERβ could also inhibit the growth of the ERα-negative ovarian cancer cell line PEO-14. These results are in agreement with our previous findings obtained with ERα-negative cell lines from breast [31] and prostate [30] cancer cells and with another study performed in ERα-negative SKOV3 EOC-derived cell line [36]. In cells devoid of estrogen receptors, it is likely that restoration of ERα cannot enable a stimulation of proliferation upon E2 treatment, since it triggers a different program of transcriptional regulation, compared to cells expressing naturally ERα, as previously showed in breast cancer cells [37]. Indeed, we observed that endogenous ERα levels in BG-1 cells were strongly reduced by the presence of ERβ, whereas ERβ affected less ERα levels in PEO-14 cells. Moreover, ERβ could inhibit completely ERα activity in BG-1 cells but not in PEO-14 cells. This could be due to the fact that in BG-1 cells, endogenous ERα is under the control of its own promoter, whereas in PEO-14 cells, exogenous ERα is controlled by a viral promoter. Moreover, one cannot exclude that the cofactors required for ERα and ERβ activity in the two cell types are different, which accounts for their differential activity in the BG-1 and PEO-14 cells.

The novelty of our study is to have extended these data to two *in vivo* models. If clinical evidences based on ERβ levels in normal tissue and cancer suggest that this receptor could act as a potential tumor suppressor, so far, no preclinical proof has been brought to confirm this hypothesis. We first used a classical subcutaneous model, to answer to this question and more precisely to determine if estrogen were required or not. BG-1 *in vivo* growth was clearly dependent on the presence of estradiol and ERβ could counteract tumor growth. We also used orthotopic implantation of bioluminescent cells in the ovary. In agreement with *in vitro* experiments, we observed a strong reduction of tumor growth when BG-1 cells express ERβ.

Cell cycle analysis demonstrated that ERβ inhibited cell proliferation, by decreasing the proportion of cells in S phase and increasing the proportion of cells in G2-M phase. This situation is similar to that reported in breast cancer cells [38]. At the molecular level, ERβ could decrease the phosphorylation of Akt and Rb. In addition, ERβ also reduced the expression of cyclin D1 and cyclin A. Cyclin D1 interacts with Cyclin-Dependent Kinase-4 and −6, which leads to the phosphorylation of Rb and the dissociation of Rb/E2F complex, which causes the progression through the G1 [35]. Cyclin A interacts with Cyclin-Dependent Kinase-2 to promote the transition from the S to G2 phase [39]. Moreover, the phosphorylation of AKT favors G1/S transition by blocking the transcriptional activity of Foxo factors, which regulate Cyclin D1 and p21 expression [40]. Based on these findings, we propose that ERβ leads to a decrease in AKT phosphorylation, which in turn results in a decreased expression of Cyclin D1 and phosphorylation of Rb.

To explain how ERβ can affect cell proliferation, we have investigated whether it could directly modify ERα expression and signaling in BG-1 cells. It is indeed interesting to notice that ERβ has also profound effects of ERα levels as it diminished by about 15 fold ERα expression in 3h. Although the exact molecular mechanisms accounting for the negative effect of ERβ on ERα in BG-1 cells remains to be elucidated, the extent and rate of degradation of ERα is certainly critical for its activity and these parameters change if ERβ is present or not. Indeed, in parental BG-1 cells, expressing only ERα, the receptor is also subjected to E2-dependent degradation. This is likely due to proteasomal degradation of the receptor as shown in a number of cell types by previous studies [41], [42]. Paradoxically, the E2-dependent proteasomal degradation of ERα is also required for its full activity through the regulation of the interactions of the receptors with its coactivators [42] and the cycling of ERα on the promoter of its target genes [43]. The situation appears different when ERβ is coexpressed with ERα in BG-1 cells. Indeed, we report that ERβ could reduce more rapidly and strongly the expression of ERα in BG-1 cells in the presence of E2, compared to cells in which only ERα is present. This is in agreement with what is observed in the ERα-positive breast cancer cell line T47-D transfected with ERβ [44]. This suggests that ERβ could trigger a rapid proteasomal degradation of ERα in the presence of E2. If we suppose that ERα and ERβ form heterodimers as previously described [45], [46], ERβ might increase the degradation of ERα present in the complex, by altering its conformation and preventing it from being active. Consequently, ERα would be less efficient to activate its target genes and could not play its proliferative role.

Indeed, in addition to its effects on ERα expression, we cannot exclude that ERβ also reduces ERα activity, as shown by transfection of an estrogen-responsive reporter in BG-1 cells. This is in agreement with a previous study performed in an ERα-positive breast cancer cell lines in which ERβ was transfected, suggesting that ERα and ERβ have distinct roles [47]. In turn, this could affect the E2-dependent signaling of ERα, which could not regulate the levels of the activity of key target genes involved in the proliferation, such as AKT, Rb, cyclin D1 or cyclin A2. Moreover, other studies have reported a negative action of ERβ on ERα signaling [44], [48]. In particular, ERβ has been shown to alter the recruitment of AP-1 complexes to ERα target genes [44], which is known to act in synergy with ERα. Once heterodimerized with ERα, ERβ could also modify the ability of ERα to interact with coactivators. A previous study has also suggested a Ying Yang action of ERβ *in vivo*, which is a repressor of ERα signaling in the presence of ERα, but can also replace ERα in the absence of ERα [48]. The down-regulation of ERα expression by ERβ may not be the only mechanism of growth inhibition by ERβ, as ERβ can also reduce cell growth of ERα-negative PEO-14 cells. It is possible that ERβ directly affect factors involved in cell proliferation, such as transcriptional coregulators, cell cycle regulators but also growth factors.

We report that ERβ could not only reduce the growth of the primary tumor, but could also decrease the extent of metastasis, or at least the presence of tumor cells in different organs. We have previously shown that ERβ could inhibit *in vitro* cancer cell invasion [31], which certainly accounts for the decreased dissemination observed. However, we cannot completely exclude the possibility that the reduced growth of the primary tumor leads also to a decreased metastasis. Whatever the effect exerted by ERβ, this reduction of metastasis certainly explains the increased survival of the mice implanted with ERβ cells.

Taken together, our findings provide evidence for a scenario in which ERβ acts as a potential tumor-suppressor and represents a potential target for future therapies of EOC. To our knowledge, this is the first *in vivo* demonstration of anti-proliferative and -metastatic actions of ERβ in a preclinical orthotopic model of ovarian carcinogenesis. Our results linking ERβ and the mestastasis process are in complete agreement with clinical studies revealing that ERβ is not expressed in metastatic forms of ovarian cancers [20] and the loss of its expression correlates with a shorter overall survival [23]. Here, we further unravel that ERβ directly impacts on ERα expression, cell cycle and invasive properties of cancer cells. The reasons accounting for the weak expression of ERβ in ovarian cancer remain elusive. Recent reports suggest possible epigenetic modifications leading to *ERβ* silencing, as treatment of ovarian cancer cells with DNA methyltransferase or histone deacetylase inhibitors could restore its expression [49], [50], [51]. Another hypothesis would be a preferential degradation of ERβ protein by the proteasome, resulting in low levels of this receptor in cancer cells [32]. These could be the tracks to explore in the future for controlling ERβ expression and developing novel therapies in ovarian cancer.

## Materials and Methods

### Tumor cell line

The human ovarian cell lines BG-1 (ERα-positive cells) [52] or PEO14 [33] were obtained from Dr. P Pujol [52] and grown in DMEM supplemented with 10% FCS and gentamycin at 37°C in a humidified atmosphere containing 5% CO_2_. To wean the cells off steroids, they were cultured in phenol red-free DMEM-F12 supplemented with 10% charcoal dextran-treated FCS (CDFCS) for 4 days.

The stably transfected BG-1-luc cell line was obtained after transfection with the plasmid CMV-LUC-Neo encoding the luciferase reporter under the control of CMV promoter. Transfected cells were then selected by G418 at a concentration of 0.5 mg/ml. Luminescent clones were identified using photon-counting camera (NightOWL II LB 983 from Berthold, France) by addition of luciferin in the growth medium, and the most responsive clones were isolated.

### RNA extraction and real-time PCR

Total RNA was isolated with TRIzol reagent (Invitrogen) as described by the manufacturer. Reverse transcription was performed using random primers and Superscript II enzyme (Invitrogen). Real-time PCR quantification was then performed using a SYBR Green approach (Light Cycler; Roche), as previously described [53]. For each sample, *ERα and ERβ* mRNA levels were normalized with *RS9* mRNA levels. The sequences of the oligonucleotides used were previously described [49].

### Recombinant adenovirus construction, propagation and infection

The non-recombinant adenovirus Ad5, and the adenovirus encoding ERα (Adα) or ERβ (Adβ) used in this study have been previously described [31]. BG-1 cells were infected overnight at a multiplicity of infection (MOI) of 25 and PEO-14 at a MOI of 100 in DMEM/F12 10% CDFCS.

### Constructs and Transient transfection

The ERE-TK-LUC construct consists of two ERE in tandem upstream of TK promoters [49]. 3.10^5^ of steroid-weaned cells were plated in 12-well plates in phenol red-free DMEM-F12, and supplemented with 10% CDFCS 24 h before transfection. Cells were infected with Ad5, Adα or Adβ viruses as mentioned above. Transfections were performed using lipofectamine according to the manufacturer's recommendations, using 2 µg of the luciferase reporter, along with 0.5 µg of the internal reference reporter plasmid (CMV-Gal) per well. After 6 h incubation, the medium was removed and the cells were placed into a fresh medium supplemented with a control vehicle (ethanol) or E2. Twenty-four hours later, cells were harvested and assayed for luciferase activity using a Centro LB960 Berthold luminometer. β-galactosidase was determined as previously described [49].

### Protein extracts and Western blots

BG-1 cells were cultured for 4 days in CDFCS. Cells were treated for 24h with 4 mM thymidine before adenoviral infection with Ad5 or Adβ viruses. Cells were harvested in Tris-glycerol buffer (Tris-HCl 50mM, EDTA 1.5mM, 10% glycerol) supplemented with protease inhibitor cocktail (Roche) and phosphatase inhibitors, and were then sonicated. 30 µg of protein extracts were subjected to SDS-PAGE protein samples Western blot analyses were done using ERα (Santa Cruz, ref SC-543), ERβ [31], Cyclin D1 (Cell Signaling, ref 2926), Akt (Cell Signaling, ref 9372), p-AKT (Cell Signaling, ref 9271), Cyclin A2 (Sigma-Aldrich, ref C0244), p-Rb (Cell Signaling, ref 9308), total Rb (Cell Signaling, ref 9309) and β-actin (Santa Cruz, ref: SC-1615) antibodies. Immunoreactivity was detected with Millipore ECL system. Actin was used as a loading control.

### Proliferation assay

20000 BG-1 or PEO14 cells were plated in 24-well plates and grown in the presence of control vehicle ethanol or 10^−8^ M estradiol for 4-days. Cells were then collected their proliferation was quantified by counting the cells on a cell counter.

### Flow cytometry

1x10^6^ BG-1 were collected 24h after infection with Ad5 or Adβ adenoviruses. Cells were resuspended in 75% ethanol and fixed for 12 min. After centrifugation, cells were incubated in PBS containing 40 µg/ml propidium iodide and 100 µg/ml RNAse for half an hour at 37°C. Cell cycle analysis was performed on an Epics-XL flow cytometer (Beckman Coulter, Fullerton, CA, USA) and analyzed with Modfit software (Verity Software, Topsham, ME, USA).

### Animal xenografts

Female Nu/Foxn1 athymic nude mice, 7 weeks old were obtained from Harlan. Mice were acclimatized for 1 week before the experiment, and were kept under pathogen-free conditions in laminar-flow boxes (5 mice/cage) maintained under standard conditions (22±2 °C, 45±10% relative humidity, 12 h light/12 h dark cycle each day, standard diet and water ad libitum). All experiments were performed in accordance with the French guidelines for experimental animal studies and declared to ethical committee (Comité d'Ethique pour l'Expérimentation Animale Languedoc Roussillon (CEEA-LR)) (Permit No. obtained for this study: CEEA-LR-11014). All efforts were made to minimize suffering.

When indicated, before cell implantation, a silicone tube (silastic) filled with a solid mixture of E2 and cholesterol as a carrier (1∶10) was implanted subcutaneously (sc) in the interscapular region of ovariectomized mice as previously described [54]. Two days later, 5.10^6^ BG-1 cells prepared in 75 µl serum-free culture medium, combined with phenol red free Matrigel (1∶1, v/v, BD Biosciences) were sc grafted on both flanks of these mice. Alternatively, 1.10^6^ BG-1 cells prepared in 20 µl serum-free culture medium combined with matrigel (2∶1, v/v) were orthotopically grafted in the left ovary surgically exposed of anaesthetized mice.

### In vivo bioluminescent imaging BG-1 cells

To measure luciferase activity, mice were first sedated by isoflurane gas anesthesia system (T.E.M., Bordeaux, France). Mice were then injected intraperitoneally with 125 mg/kg body weight of luciferin (sodium salt; Promega) in aqueous solution. Luminescence was measured using NightOWL II LB 981 CCD camera and integrated for a 5-min period. The signal intensities from regions of interest (ROI) were obtained and data were expressed as photon (Ph/s). Background was defined from a region of the same size placed in a non-luminescent area nearby the animal and then subtracted from the measured luminescent signal intensity. The correlation of luciferase signal with tumor volume and weight was demonstrated (Figure S1).

### Tissues extracts luciferase activity

Lysates from tissue samples were prepared in ceramic beads-containing tubes (Lysing matrix, MP Biomedicals), by disruption in luciferase lysis buffer (25mM Tris Phosphate pH7.8, 2 mM DTT, 2 mM EDTA, 10% Glycerol, 1% Triton X100, 1mg/ml BSA). The samples were subjected to two oscillations at 7,000 r/min for 15 seconds. The lysates were then centrifuged at 10,000 rpm for 30 min at 4°C, and the supernatant was saved and assayed. 10 µl of the supernatant were loaded onto 96-well white opaque tissue plates (Lumitrac 200), and luciferase activity was measured as previously described [49].

## Supporting Information

Figure S1 **In vivo monitoring of orthotopically injected BG-1-luc cells in the left ovary.** Cells were injected into the bursal membrane of the left ovary and animals were monitored by bioluminescence. At day 25, animals were euthanized, and bioluminescence, the volume and weight of the ovary were measured. Correlation of the volume of the tumor (left panel) or weight (right panel) with the luciferase is shown.
(TIF)

Figure S2 **In vitro growth of PEO14 cells expressing or not ERβ.** In vitro growth was monitored by counting the cells on a cell counter after 4 days of proliferation. PEO14 cells were infected with Ad5, Adα or Adβ virus and cultured in the presence of control vehicle ethanol (Control) or E2 (10–8M). Proliferation is expressed as fold of control cells grown at day 4. Data represent the mean ± SD from triplicates. Measurements of Adα and Adβ groups were compared to Ad5 by unpaired Student's t test. Only Adβ groups were significantly different from Ad5 groups.
(TIF)
