# Correction: Potential Role of Estrogen Receptor Beta as a Tumor Suppressor of Epithelial Ovarian Cancer

**DOI:** 10.1371/annotation/480acc26-456b-4e06-8cb6-2834bd6f5553

**Published:** 2013-05-10

**Authors:** Carine Bossard, Muriel Busson, David Vindrieux, Françoise Gaudin, Véronique Machelon, Madly Brigitte, Carine Jacquard, Arnaud Pillon, Patrick Balaguer, Karl Balabanian, Gwendal Lazennec

There was an error in the Funding statement.

The correct statement is: This work was supported by grants from ARC (Association pour la Recherche contre le Cancer, Grant No. 3582) (http://www.arc-cancer.net), The Ligue Nationale Contre le Cancer (http://www.ligue-cancer.net). Muriel Busson was supported by FRM (Fondation pour la Recherche Médicale) (http://www.frm.org). DV was a recipient of the Ligue Nationale contre le Cancer. CB was supported by a HFSP fellowship (http://www.hfsp.org/). The K. B. lab is supported by the Agence Nationale de la Recherche (ANR, grant number 2010 JCJC 1104 01) (http://www.agence-nationale-recherche.fr ​/), the Université Paris-Sud, the Institut National de la Santé et de la Recherche Médicale (INSERM) and the Ligue Nationale Contre le Cancer (Comité Val d'Oise), and is a member of the Laboratory of Excellence LERMIT (Laboratory of Excellence in Research on Medication and Innovative Therapeutics) supported by a grant from ANR (Investissements d´Avenir). Dr Madly Brigitte was supported by the Fondation ARC and Dr Carine Jacquard by INCA (Institut National du Cancer). The funders had no role in study design, data collection and analysis, decision to publish, or preparation of the manuscript. 

