# Supplementary figures and images for: Potential Role of Estrogen Receptor Beta as a Tumor Suppressor of Epithelial Ovarian Cancer
Source: PLoS One. 2012 Sep 6;7(9):e44787. doi: 10.1371/journal.pone.0044787 (PMC3435304; doi:10.1371/journal.pone.0044787)

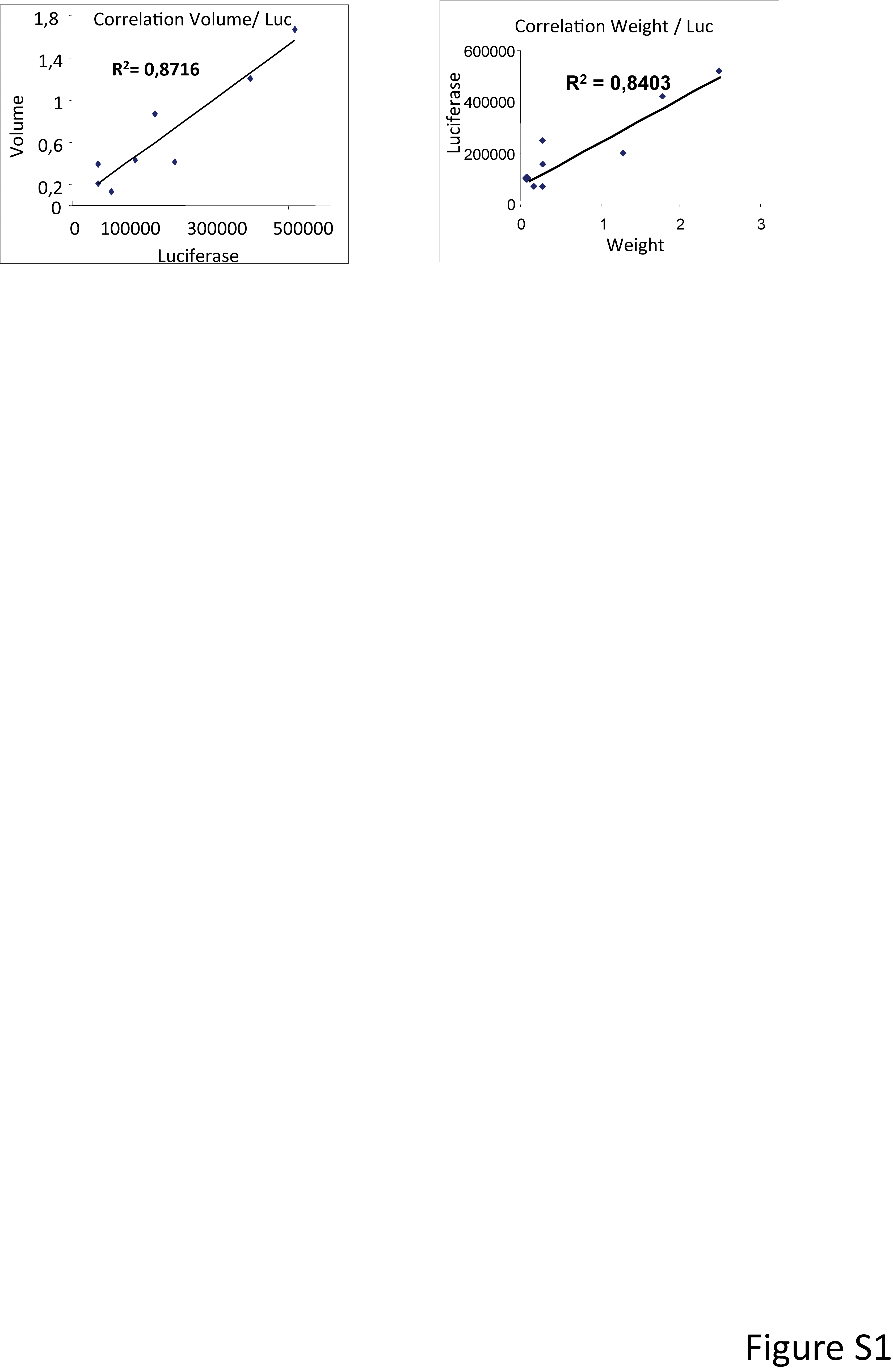

Supplement: Figure S1 — In vivo monitoring of orthotopically injected BG-1-luc cells in the left ovary. Cells were injected into the bursal membrane of the left ovary and animals were monitored by bioluminescence. At day 25, animals were euthanized, and bioluminescence, the volume and weight of the ovary were measured. Correlation of the volume of the tumor (left panel) or weight (right panel) with the luciferase is shown. (TIF) [file pone.0044787.s001.tif]

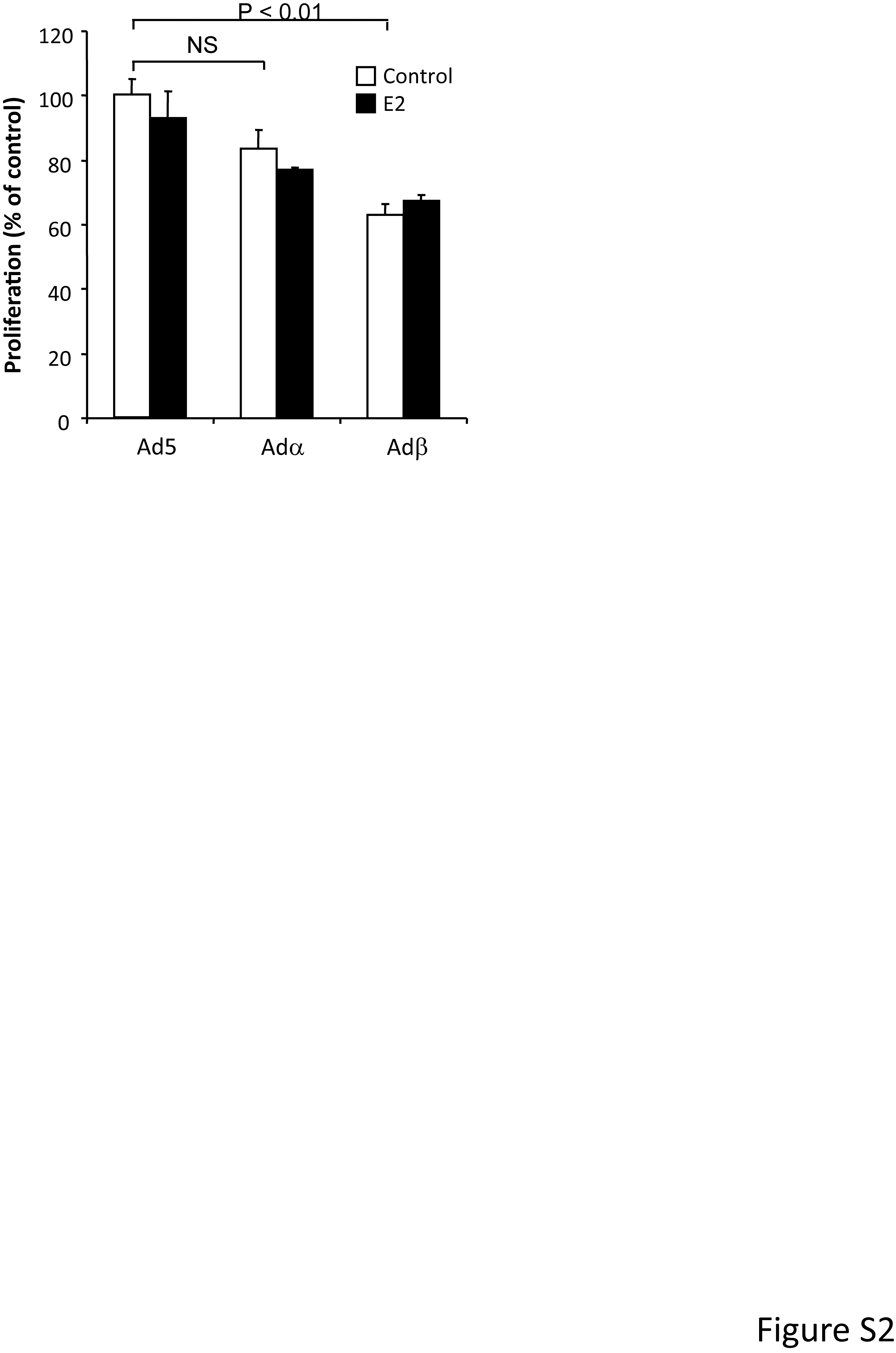

Supplement: Figure S2 — In vitro growth of PEO14 cells expressing or not ERβ. In vitro growth was monitored by counting the cells on a cell counter after 4 days of proliferation. PEO14 cells were infected with Ad5, Adα or Adβ virus and cultured in the presence of control vehicle ethanol (Control) or E2 (10–8M). Proliferation is expressed as fold of control cells grown at day 4. Data represent the mean ± SD from triplicates. Measurements of Adα and Adβ groups were compared to Ad5 by unpaired Student's t test. Only Adβ groups were significantly different from Ad5 groups. (TIF) [file pone.0044787.s002.tif]
